# Kalirin-7 plays a neuroprotective role in Neuro-2A cells injured by oxygen-glucose deprivation and reperfusion through Rac1 activation

**DOI:** 10.22038/IJBMS.2018.28523.6916

**Published:** 2018-10

**Authors:** Jian Xu, Yanxiu Chen, Zeyu Wu, Yihe Dou, Peng Lun, Peng Sun

**Affiliations:** 1 Department of Neurosurgery, The Affiliated Hospital of Qingdao University, Qingdao 266500, China; 2 Department of Neurology, Liaocheng People’s Hospital, Liaocheng 252000, China

**Keywords:** Anti-apoptosis, Kalirin-7, Neuroprotection, OGD/R, Rac1

## Abstract

**Objective(s):**

The study explored the neuroprotective role of Kalirin-7 (Kal-7) in Neuro-2A cells after oxygen-glucose deprivation and reperfusion (OGD/R) treatment.

**Materials and Methods:**

The study used an OGD/R model of mouse Neuro-2A neuroblastoma cells *in vitro*. Cells were transfected with pCAGGS-Kal-7 to up-regulating kal-7. Then cell proliferation and apoptosis were respectively analyzed by Trypan blue exclusion method and flow cytometry. To examine the involvement of Rac1, cells were treated with Rac1-GTP inhibitor NSC23766 before treatment with OGD/R. Expressions of Bax, Bcl-2, Rac1, and down-stream targets of Rac1 were analyzed by Western blot.

**Results:**

Kal-7 significantly decreased OGD/R induced cell apoptosis (*P<*0.01), but no significant effects were observed on cell proliferation. Kal-7 increased the expressions of apoptosis-related protein of Bcl-2 and Rac1, but decreased the expression of Bax in Neuro-2A cells stimulated to OGD/R. Rac1 was activated by Kal-7 due to the increased levels of its down-stream targets, p-p38 and p-PAK1. NSC23766 reduced the anti-apoptotic effect of Kal-7 as the enhanced apoptotic cell rate and increased Bax/Bcl-2 ratio.

**Conclusion:**

These findings suggest that the protective effects of Kal-7 against OGD/R injury in Neuro-2A cells were dependent in a Rac1 activation signaling.

## Introduction

Kalirin is an important member of the Dbl family, which is a Rho guanine nucleotide exchange factor (GEF), and is named as multiple-handed Hindu goddess Kali according to the interact ability with various other proteins (1, 2). Neuronal GEF kalirin is a key regulator of dendritic spine structure and functional plasticity, as well as shows a crucial role in nerve growth and axonal development (1-3). Recently, increasing researches have proven the colsely links between kalirin signaling and various disorders, such as schizophrenia and Alzheimer’s disease (4, 5). Kalirin signaling may help to understand pathogenesis of several neurological diseases and thus provide a novel therapeutic target (4). 

Kalirin-7 (Kal-7), the most common and major isoform of kalirin family, which exerts in the adult rodent brain, especially in the synaptic side of the excitatory postsynaptic synapse (2, 6-8). It is generally known that Kal-7 is a polyhedral molecule, which contains several fileds that can interact with a wide range of molecular machinery (9). Kal-7 is a GEF of small GTPases family, which is usually used to mediate the formation and maintenance of dendrites and spines (6, 10, 11). Kal-7 activation can lead to the formation of mature dendritic spines by binding to the ephrin of the EphB2 receptor, which in turn activates Rac and PAK1 (11). According to previous studies, Kal-7 is essential for synapse remodeling in mature cortical neurons, and is thought to play a vital role in the pathogenesis of schizophrenia (1, 2). 

Recent study demonstrated that overexpression of Kal-7 could induce the great number of aberrant spine-like structures formation, dependenting on its GEF activity. Moreover, inhibition of kalirin could decline the density of spines as well as decrease the loss of pre-synaptic and post-synaptic markers (11). Above evidences provide an important pathway by which activation of transsynaptic ephrin-EphB receptor leads to the Rac-mediated actin reorganization.

Neurons in the affected areas generally have shortage of oxygen and glucose, which subsequently leads to neuronal loss in the central necrosis area and cellular stress injury in the penumbral region of brain infarction (12). Therefore, an oxygen-glucose deprivation and reperfusion (OGD/R) model mimicing the pathological changes of mouse neocortical cell cultures has become an* in vitro* model in studies on nerve injury (12). It has been reported that Kal-7 may have a neuroprotective effect during inflammation of the central nervous system through inhibition of inducible nitric-oxide synthase (iNOS) activity (13). But the role of Kal-7 in neuroprotection in OGD/R model and the underlying mechanism of Kal-7 that mediates neuroprotection against OGD/R injury through Rac1 are still unclear.

Hence, the study is meant to explore the effect of Kal-7 on Neuro-2A cells exposed to OGD/R. The regulatory effect of Kal-7 on cell apoptosis was studied and the related mechanism in neuroprotection of Neuro-2A cells was also explored. 

## Materials and Methods

***Cell culture***

Neuro-2A, a mouse neuroblastoma cell line, was purchased from American Type Culture Collection (Manassas, VA, USA). The cell cryopreservation tube was took out from the liquid nitrogen container, and directly immersed the tube in the water at 37 ^°^C to melt the cells. Then, cell cryopreservation tube was opened on the sterile super clean workbench, and the cell suspension was sucked with a straw and added to a centrifuge tube. After centrifugation for 5 min, the supernatant was discarded, and cells were re-suspended with 10% fatal bovine serum (FBS), and were then transferred to a new culture bottle. The minimum essential medium (MEM; GIBCO BRL, Grand Island, NY, USA) containing 10% FBS, L-glutamine, 100 IU/ml penicillin, and 100 μg/ml streptomycin was added to the flasks, and these cells were cultured in a incubator containing 5% CO_2_ at 37 ^°^C.

***Plasmid construction and transfetcion assay***

The cDNA of Kal-7 was obtained from PCR amplification by using a plasmid containing the wild type Kal-7 gene. The PCR product was cloned into the pCAGGS-EGFP vector. Then, the pCAGGS-EGFP and pCAGGS-Kal-7 vectors were transfected into Neuro-2A cells by using Lipofectamine 2000 (Invitrogen, CA, USA) as described by the manufacturer. After transfection for 48 hr, cells were harvested for using in the subsequent experiments.

***Oxygen-glucose deprivation and reperfusion (OGD/R) procedure***

A model of OGD/R-induced Neuro-2A cell injury was contructed as described previously (14). Mouse Neuro-2A neuroblastoma cells in the OGD/R group were washed with glucose-free Earle’s balanced salt solution (EBSS, Invitrogen) for three times, and incubated in an anaerobic chamber containing oxygen-free 5% CO_2_ and 95% N_2_ at 37 ^°^C. After incubation for 4 hr, the treated Neuro-2A cells were then returned to the standard culture medium, and incubated for another 12 hr for recovery under the normoxic culture conditions (5% CO_2_ and 95% O_2_). Cells were treated with EBSS containing glucose for 4 hr and incubated in the normal medium as a control group.

***Determination of the role of Rac1 in neuroprotection***

To investigate whether Rac1 was participated in the mediation of neuroprotective effect of Kal-7 on OGD/R-induced Neuro-2A cells, the different cultures were pretreated with NSC23766 (a Rac1-GTP inhibitor). NSC23766 was dissolved in 0.2% dimethyl sulfoxide (DMSO, Invitrogen) to a concentration of 10 μM and then Neuro-2A cells were incubated with NSC23766 for 30 min before they were subjected to OGD/R.

***Cell proliferation assay***

Cell proliferation was evaluated by 0.4% trypan blue exclusion cell counting method.

Total of about 8.0 × 10^4^ cells were plated in 24-well plates and treated with pCAGGS-EGFP and pCAGGS-Kal7. Cell counts were conducted by trypan blue staining after 1, 2 or 4 hr. The experiment was repeated 3 times. 

***Flow cytometry analysis***

After transfection and treatment, Neuro-2A cells were harvested and washed with pre-cold PBS for 2 times. Then, these cells were re-suspended in 1 × binging buffer, and 100 μl cell suspension was added to the bottom of flow tube. Subsequently, 5 μl Annexin V-FITC and 5 μl of propidium iodide (PI) (Invitrogen) were also added to the flow tube and stained Neuro-2A cells for 15 min under the condition of room temperature shading. After staining, cells were re-suspended in 400 μl 1 × binging buffer, and the percent of apoptotic cells was assessed by fluorescence-activated cell sorting (FACS) with a BectonDickinson FACScan (Immunocytochemistry Systems, San Jose, CA, USA). Viable cells were labeled as Annexin V- and PI-, early apoptotic cells are labeled as Annexin V+ and PI-, necrotic cells are marked as Annexin V- and PI+, and the late apoptotic cells are marked as Annexin V+ and PI+.

**Figure 1:**
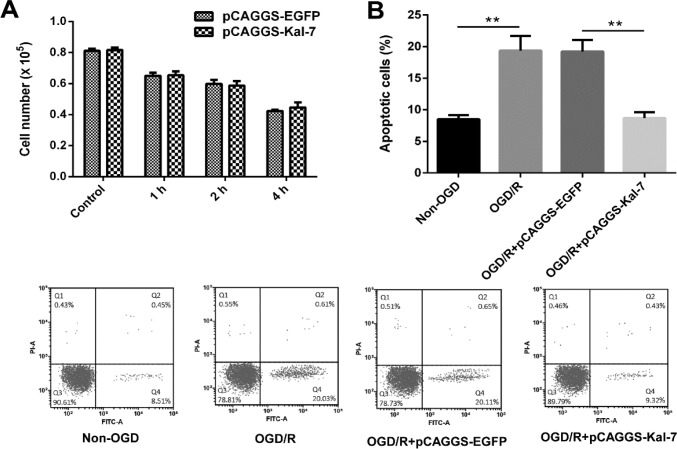
Effects of Kal-7 on cell proliferation and cell apoptosis. 1A: Kal-7 showed no significant effect on cell proiliferation. 1B: Kal-7 showed a significant decrease in OGD/R-induced cell apoptosis **P<0.01

**Figure 2:**
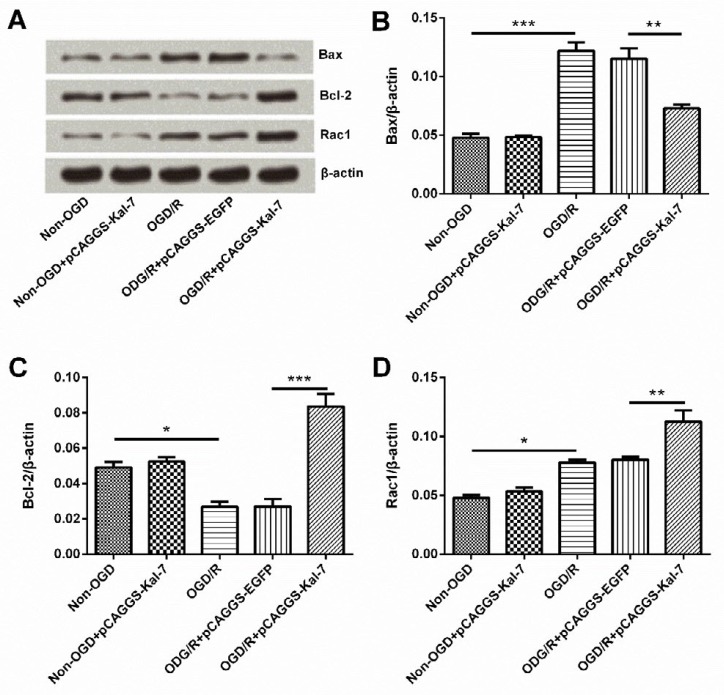
Effects of Kal-7 on expression of apoptosis-related proteins. 2A: Results of Western blot analysis of Kal-7 on expressions of apoptosis-related proteins. 2B: Effects of Kal-7 on expression of Bax. 2C: Effects of Kal-7 on expression of Bcl-2. 2D: Effects of Kal-7 on expression of Rac1. *P<0.05, **P<0.01, and ***P<0.001

**Figure 3:**
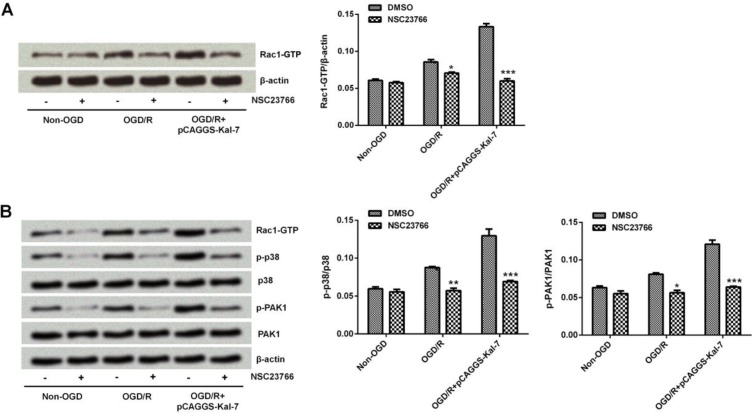
Effects of NSC23766 on Rac1 activation and its downstream target expression. 3A: Effects of NSC23766 on expression of Rac1-GTP. 3B: Effects of NSC23766 on expressions of downstream targets of Rac1. *P<0.05, **P<0.01, and ***P<0.001

**Figure 4:**
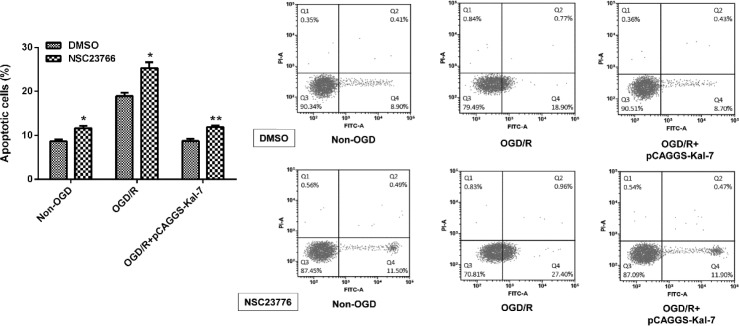
Effect of NSC23766 on protective effect of Kal-7 against OGD/R induced apoptosis. *P<0.05 and **P<0.01

**Figure 5:**
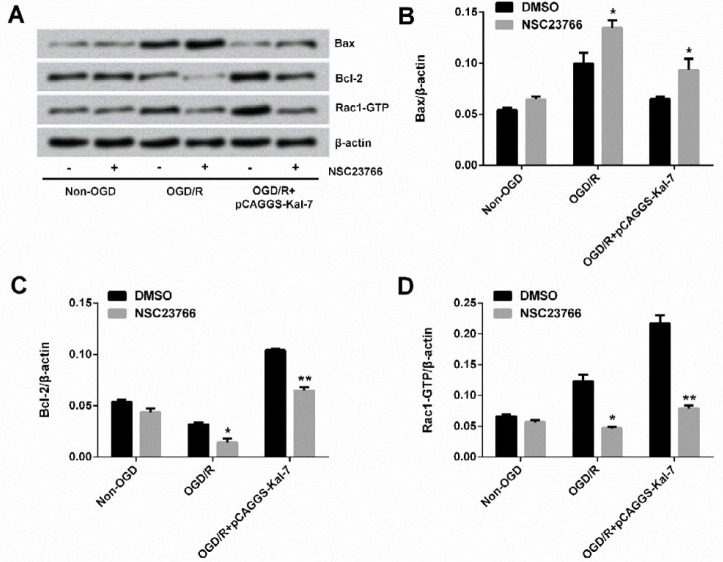
Effect of NSC23766 on anti-apoptotic effect of Kal-7. 5A: Results of Western blot analysis of NSC23766 on expressions of apoptosis-related proteins. 5B: Effects of NSC23766 on expression of Bax. 5C: Effects of NSC23766 on expression of Bcl-2. 5D: Effects of NSC23766 on expression of Rac1-GTP. *P<0.05 and **P<0.01

***Western blot***

Cells from different groups were lysed in radioimmunoprecipitation assay (RIPA) buffer (Beyotime Biotechnology, Shanghai, China) for whole cell protein preparations. The BCA™ Protein Assay Kit (Pierce, Appleton, WI, USA) was used to measure the total protein concentration, and the equal proteins were electrophoresed by 10% sodium dodecyl sulfate polyacrylamide gel electrophoresis (SDS-PAGE). After the end of electrophoresis, these proteins were transferred to PVDF membranes and were blocked with 5% BSA in Tris-buffered saline with Tween 20 (TBST). Subsequently, the PVDF membranes were incubated with the diluted primary antibodies for 12 hr, and then were shake-washed with TBST for 30 min at room temperature. After incubation and washing, the PVDF membranes were incubated with secondary antibody for another 1 hr. The following primary antibodies of rabbit monoclonal anti-Bax (ab32503, predicted molecular weight: 21 kDa), rabbit polyclonal anti-Bcl-2 (ab59348, predicted molecular weight: 26 kDa), rabbit polyclonal anti-Rac1 (ab155938, predicted molecular weight: 21 kDa), rabbit polyclonal anti-p-p38 (ab47363, predicted molecular weight: 41 kDa), rabbit monoclonal anti-p38 (ab170099, predicted molecular weight: 42 kDa), rabbit polyclonal anti-p-PAK1 (ab75599, predicted molecular weight: 61 kDa), and rabbit monoclonal anti-PAK1 (ab40852, predicted molecular weight: 66 kDa, Abcam, San Francisco, USA) were used at a dillution of 1:1000. The second antibody of goat anti-rabbit IgG conjugated with horseradish peroxidase (ab205718, Abcam) was used at a dillution of 1:500. β-actin (rabbit polyclonal, ab8227, predicted molecular weight: 42 kDa) was used as an internal control. The ECL Western blotting reagent were used to present the bindings, and the experiment Western blot bands were analyzed by using Image J Software (version 1.41).

***Statistical analysis***

All data from this study was expressed as mean+ SD. for three different determinations. The SPSS/Win 17.0 software (SPSS Inc., Chicago, IL, USA) was used for statistical analysis. Statistical significance between two groups was analyzed by student’s *t* test, and statistical significance of multiple was analyzed by one-way analysis of variance (ANOVA). *P*<0.05, *P*<0.01, and *P<*0.001 were considered as statistically significant. 

## Results

***Effects of Kal-7 on cell proliferation and apoptosis***

The proliferation and apoptosis of Neuro-2A cells transfected with pCAGGS-EGFP and pCAGGS-Kal-7 were respectively determined by Trypan blue exclusion method and flow cytometry method. As shown in Figure 1A, Kal-7 did not significantly alter the proliferation of Neuro-2A cells at all tested time points compared to control group. Flow cytometry analysis revealed that OGD/R induced significant decrease in cell apoptosis relative to non-OGD cells (*P<*0.01). Compared with pCAGGS-EGFP group, apoptosis of cells in the PCAGGS-Kal-7 group was significantly suppressed (*P<*0.01, Figure 1B).

***Effects of Kal-7 on expressions of apoptosis-related proteins and Rac1***

Results revealed that OGD/R treatment prominently promoted the expressions of Bax and Rac1 (*P*<0.001 and *P*<0.05) but decreased the expression level of Bcl-2 (*P*<0.05). Kal-7 almost had no effect on expressions of apoptosis-related proteins in normal cells but alleviated OGD/R-induced effects by decreasing Bax expression (*P*<0.01) and elevating Bcl-2 expression (*P*<0.001). Interestingly, Kal-7 also significantly enhanced Rac1 expression (*P*<0.01). According to results displayed in Figure 2, we found that Kal-7 inhibited apoptosis of Neuro-2A cells exposed to OGD/R through Bax/Bcl-2 imbalance. Results also indicate that the changes of Bax and Bcl-2 might be correlated with the abnormal expression of Rac1.

***Kal-7 enhanced Rac1 activity and further affected its down-stream targets in OGD/R treated cells***

As described above, Rac1 was up-regulated after Neuro-2A cells were administrated with OGD/R, and was further increased by pCAGCS-Kal-7 transfection, indicating Rac1 might be involved in the neuroprotective effect of Kal-7. Next, cells were pretreated with Rac1-GTP inhibitor, NSC23766 and Western blot analysis was used to determine the role of Rac1 in OGD/R-treated cells by detecting Rac1-GTP content as well as the activation of down-stream targets of Rac1. Rac1-GTP expression was significantly suppressed by NSC23766 (*P*<0.001, Figure 3A). The p-p38 and p-PAK1 levels were increased after OGD/R treatment and further increased by Kal-7. When Rac1 activity was inhibited by NSC23766, p-p38 and p-PAK1 expressions were all reduced (*P*<0.001, Figure 3B). These data indicate that Kal-7 enhanced Rac1 activity, which then activated expressions of its down-stream targets. 

***Rac1 activation was involved in the anti-apoptosis role of Kal-7***

To further determine whether the activation of Rac1 was contributed to Kal-7 mediated neuroprotection against OGD/R, cells were treated with NSC23766 and DMSO-treated cells were used as controls. The percentage of apoptotic cells was evaluated by flow cytometric analysis. After Rac1 activity was depressed by NSC23766, the apoptotic cell rate showed a significant increase in the different processing groups of non-OGD/R, OGD/R and OGD/R+pCAGGS-Kal-7 compared to their respective control (*P*<0.05, *P*<0.05, and *P*<0.01, Figure 4). Thus we can infer that Rac1 activation participated in the anti-apoptotic role of Kal-7.

***Rac1 activation affected the regulatory effect of Kal-7 on expressions of apoptosis-related proteins***

Bax and Rac1-GTP were increased but Bcl-2 was decreased after OGD/R treatment relative to non-OGD control; whereas when inhibition of Rac1 activation was induced by NSC23766, Bax expression level was further increased (*P*<0.05), Bcl-2 expression level was further decreased (*P*<0.05), and Rac1-GTP expression level was diminished (*P*<0.05)(Figure 5). As described previously, Kal-7 was notably down-regulated Bax expression, and up-regulated Bcl-2 and Rac1-GTP expressions. Now, we found that NSC23766 treatment exhibited the contrary effects on expressions of them, inducing up-regulation of Bax (*P*<0.05) and down-regulation of Bcl-2 and Rac1-GTP (both *P*<0.01). These results suggest that Kal-7 might protect Neuro-2A cells against OGD/R injury via reducing apoptosis by activating Rac1.

## Discussion

Our study demonstrated that Kal-7 played an important neuroprotective role in neurons suffered from OGD/R-induced damage. Kal-7 could effectively inhibit OGD/R-caused apoptosis. Next, we found that Kal-7 exerted the protective effects by enhancing the activation of Rac1. In order to further explore the mechanism, cells were administrated with Rac1-GTP inhibitor, NSC23766, cell apoptosis were then detected. 

Previous researches have proven that Kal-7 is necessary for the formation and maintenance of dendritic spines (2, 6). Kal-7 showed no significant effects on cell proiliferation but significantly decreased the OGD/R-induced apoptosis of Neuro-2A cells. Kal-7 has been reported to participate in the regulation of ischemic signal transduction, and also exert neuroprotective effect during inflammation in the central nervous systems (13, 15). These findings were consistent to the findings of our study.

Kal-7 showed a significant increase in the protein level of Bcl-2 but decreased the protein level of Bax. These results demonstrated that Kal-7 could alleviate OGD/R-induced high expression of Bax and low expression of Bcl-2. Besides, high amount of Rac1 was found in OGD/R-treated cells and also found in Kal-7 over-expressed cells. Rac1-GTP exhibited the same changes. Rac1 improvement might be a protective reaction that partially reduced the injury of OGD/R. These changes suggest that Bax and Bcl-2 may be correlated with the activity of Rac1 and we doubt that Kal-7 may regulate apoptosis through Rac1 pathway. Increasing evidences showed that Rac1 could activate both pro-apoptotic and anti-apoptotic pathways (16). Ferri *et al.* reported the protective roles of Rac1 activation in endothelial cells in vascular diseases (17) and also in UV-light-induced skin carcinogenesis and keratinocyte apoptosis (18). Oppositely, there is a study demonstrating that Rac1 silence could ameliorate neuronal oxidative stress damage by diminishing Bcl-2/Rac1 complex (19). Thus, the role of Rac1 activation was further explored in our study.

Rac1 is required for activation of PAK1 and p38 (20, 21) and thereby PAK1 and p38 expressions were detected to assess the activation of Rac1 in this paper. The level phosphorated PAK1 and p38 exhibited the similar changes with Rac1-GTP, indicating that Rac1 was indeed activated by Kal-7. We then speculated that the activation of Rac1 regulated by Kal-7 played an activity of neuroprotection. To confirm this speculation, a specific inhibitor of Rac1-GTP, NSC23766, was used in the present experiment. We found that Rac1-GTP inhibitor NSC23766 significantly increased the apoptosis of OGD/R and OGD/R+Kal-7 groups, and thereby attenuating Kal-7-mediated protection against OGD/R injury. Above evidences suggest that the protective effects of Kal-7 are indeed exerted via activation of Rac1. 

Actually, Kal-7 was as an up-stream activator of Rac1 (1), which explained very well the up-regulation of Rac1 and Rac1-GTP in Kal-7-overexpressed cells. In neural cells, it has reported that Kal-7 exerts the anti-apoptotic and pro-survival effects might through activation of Rac1 signaling (22). Our study found that Kal-7-Rac1 protected against apoptosis through up-regulation of Bcl-2 and down-regulation of Bax in OGD/R-injured Neuro-2A cells, indicating that Rac1 signaling is probably involved in Kal-7 mediated neuroprotection by regulating Bax and Bcl-2. Interestingly, one previous study showed that Rac1 was considered as a novel binding partner of Bcl-2 and stabilized its anti-apoptotic activity (23), which was not contradictory with our data.

## Conclusion

Results of the present study suggest that Rac1 signaling acted as a vital regulator in the mediation of the neuroprotective effects of Kal-7. The activation of Rac1 might contribute to up-regulate Bcl-2 expression and down-regulate Bax expression in OGD/R-injured Neuro-2A cells. The decreased Bax/Bcl-2 ratio was consequently associated with the inhibition of apoptosis of Neuro-2A cells. These results suggest that up-regulating Kal-7 may provide a new gene therapy in neurological diseases and injuries by activating the downstream pathway of Rac1.

## Competing Interests

The authors declare that they have no competing interests.
